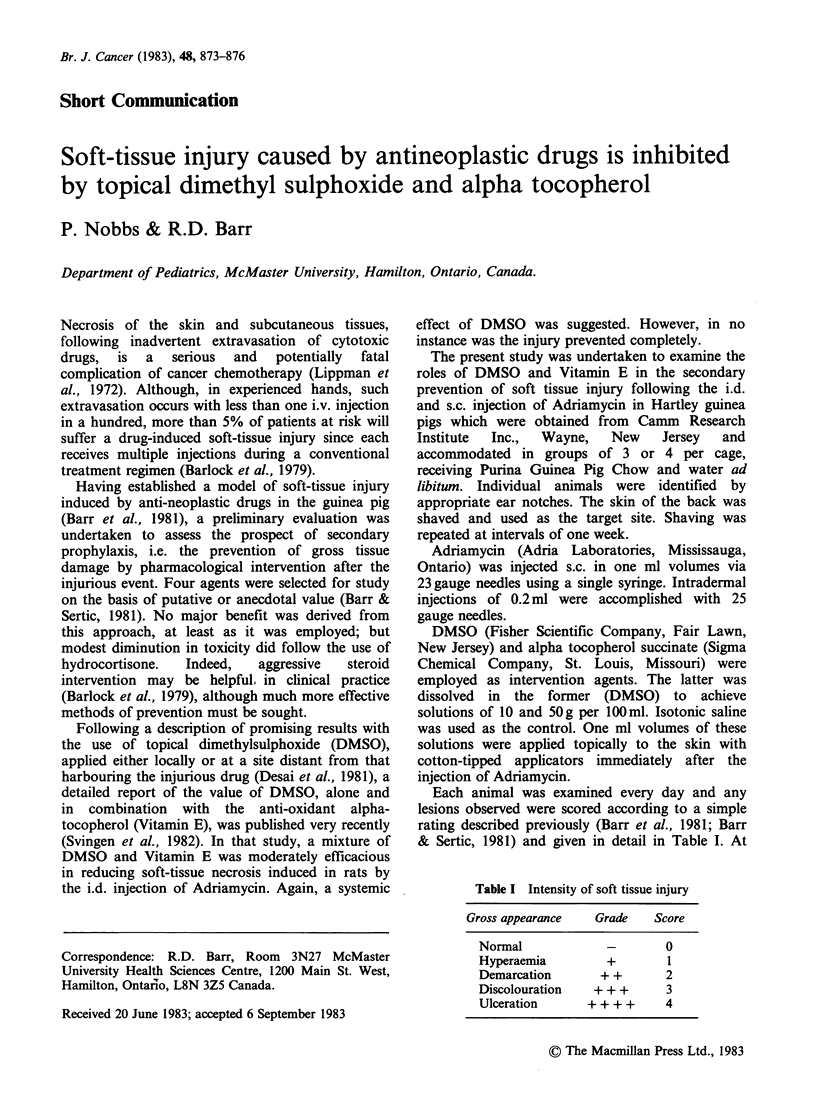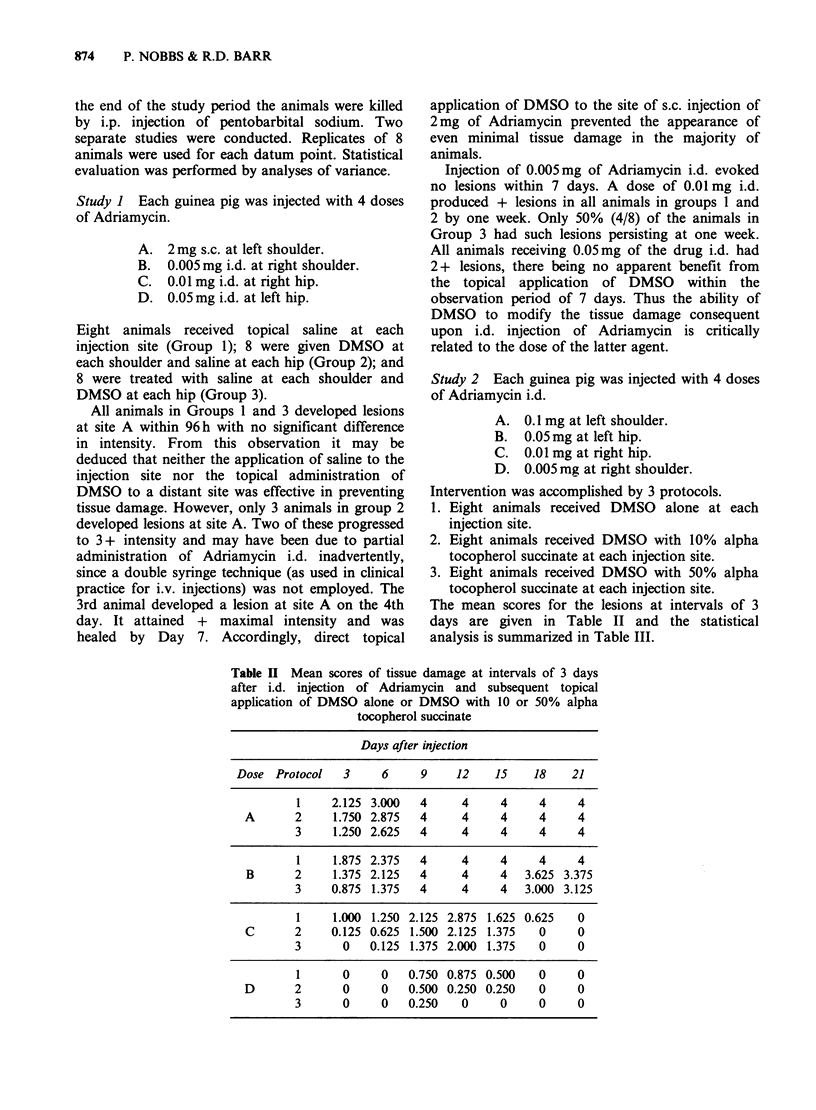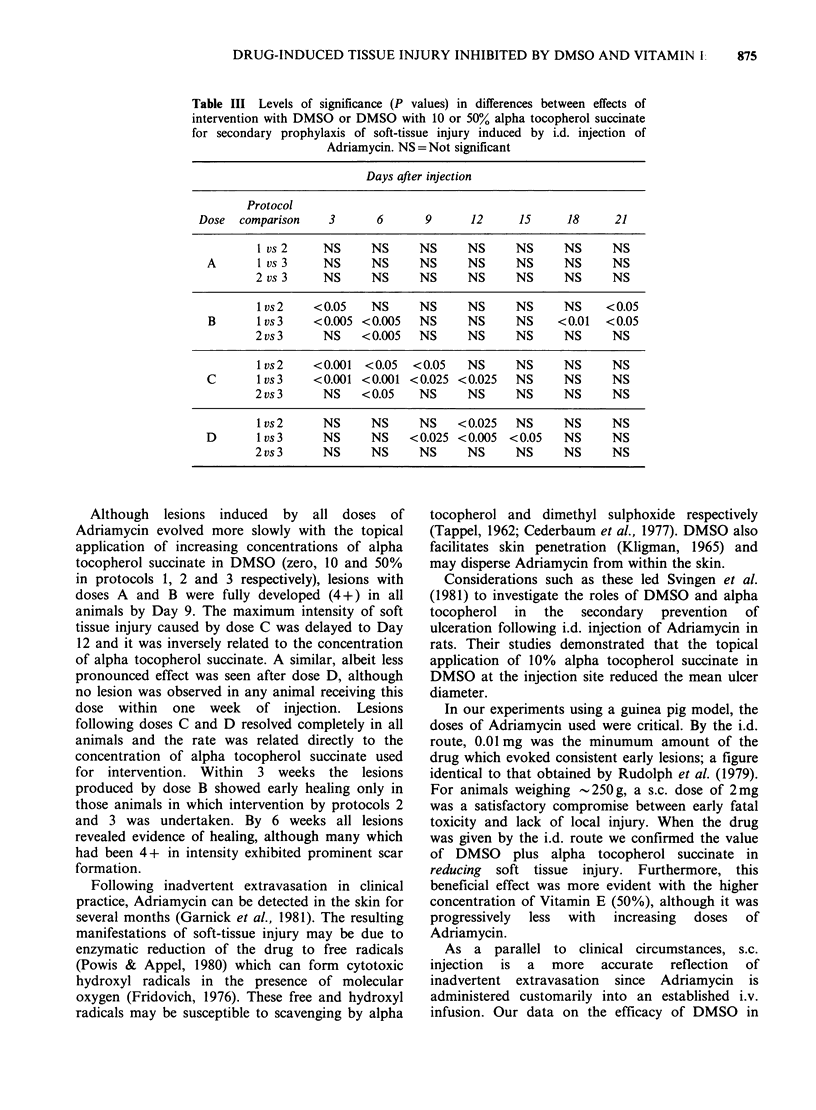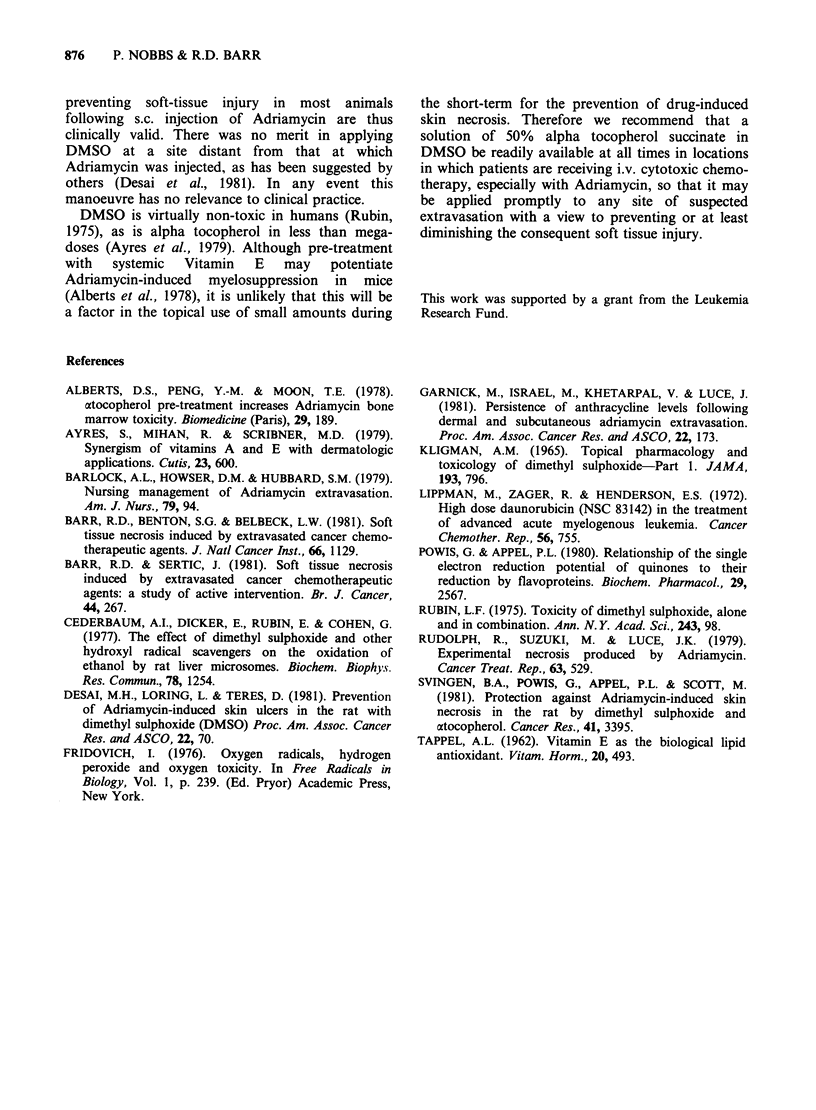# Soft-tissue injury caused by antineoplastic drugs is inhibited by topical dimethyl sulphoxide and alpha tocopherol.

**DOI:** 10.1038/bjc.1983.281

**Published:** 1983-12

**Authors:** P. Nobbs, R. D. Barr


*Br. J. Cancer* (1983), **48**, 873–876

### Short Communication

### Soft-tissue injury caused by antineoplastic drugs is inhibited by topical dimethyl sulphoxide and alpha tocopherol

P. Nobbs & R.D. Barr

*Department of Pediatrics, McMaster University, Hamilton, Ontario, Canada.*

Necrosis of the skin and subcutaneous tissues, following inadvertent extravasation of cytotoxic drugs, is a serious and potentially fatal complication of cancer chemotherapy (Lippman *et al.*, 1972). Although, in experienced hands, such extravasation occurs with less than one i.v. injection in a hundred, more than 5% of patients at risk will suffer a drug-induced soft-tissue injury since each receives multiple injections during a conventional treatment regimen (Barlock *et al.*, 1979).

Having established a model of soft-tissue injury induced by anti-neoplastic drugs in the guinea pig (Barr *et al.*, 1981), a preliminary evaluation was undertaken to assess the prospect of secondary prophylaxis, i.e. the prevention of gross tissue damage by pharmacological intervention after the injurious event. Four agents were selected for study on the basis of putative or anecdotal value (Barr & Sertic, 1981). No major benefit was derived from this approach, at least as it was employed; but modest diminution in toxicity did follow the use of hydrocortisone. Indeed, aggressive steroid intervention may be helpful in clinical practice (Barlock *et al.*, 1979), although much more effective methods of prevention must be sought.

Following a description of promising results with the use of topical dimethylsulphoxide (DMSO), applied either locally or at a site distant from that harbouring the injurious drug (Desai *et al.*, 1981), a detailed report of the value of DMSO, alone and in combination with the anti-oxidant alpha-tocopherol (Vitamin E), was published very recently (Svingen *et al.*, 1982). In that study, a mixture of DMSO and Vitamin E was moderately efficacious in reducing soft-tissue necrosis induced in rats by the i.d. injection of Adriamycin. Again, a systemic effect of DMSO was suggested. However, in no instance was the injury prevented completely.

The present study was undertaken to examine the roles of DMSO and Vitamin E in the secondary prevention of soft tissue injury following the i.d. and s.c. injection of Adriamycin in Hartley guinea pigs which were obtained from Camm Research Institute Inc., Wayne, New Jersey and accommodated in groups of 3 or 4 per cage, receiving Purina Guinea Pig Chow and water *ad libitum*. Individual animals were identified by appropriate ear notches. The skin of the back was shaved and used as the target site. Shaving was repeated at intervals of one week.

Adriamycin (Adria Laboratories, Mississauga, Ontario) was injected s.c. in one ml volumes via 23 gauge needles using a single syringe. Intradermal injections of 0.2 ml were accomplished with 25 gauge needles.

DMSO (Fisher Scientific Company, Fair Lawn, New Jersey) and alpha tocopherol succinate (Sigma Chemical Company, St. Louis, Missouri) were employed as intervention agents. The latter was dissolved in the former (DMSO) to achieve solutions of 10 and 50 g per 100 ml. Isotonic saline was used as the control. One ml volumes of these solutions were applied topically to the skin with cotton-tipped applicators immediately after the injection of Adriamycin.

Each animal was examined every day and any lesions observed were scored according to a simple rating described previously (Barr *et al.*, 1981; Barr & Sertic, 1981) and given in detail in Table I. At

**Table I** Intensity of soft tissue injury

| *Gross appearance* | *Grade* | *Score* |
|---|---|---|
| Normal | – | 0 |
| Hyperaemia | + | 1 |
| Demarcation | ++ | 2 |
| Discolouration | +++ | 3 |
| Ulceration | ++++ | 4 |

Correspondence: R.D. Barr, Room 3N27 McMaster University Health Sciences Centre, 1200 Main St. West, Hamilton, Ontario, L8N 3Z5 Canada.

Received 20 June 1983; accepted 6 September 1983

© The Macmillan Press Ltd., 1983

the end of the study period the animals were killed by i.p. injection of pentobarbital sodium. Two separate studies were conducted. Replicates of 8 animals were used for each datum point. Statistical evaluation was performed by analyses of variance.

*Study 1* Each guinea pig was injected with 4 doses of Adriamycin.

A. 2 mg s.c. at left shoulder.
B. 0.005 mg i.d. at right shoulder.
C. 0.01 mg i.d. at right hip.
D. 0.05 mg i.d. at left hip.

Eight animals received topical saline at each injection site (Group 1); 8 were given DMSO at each shoulder and saline at each hip (Group 2); and 8 were treated with saline at each shoulder and DMSO at each hip (Group 3).

All animals in Groups 1 and 3 developed lesions at site A within 96 h with no significant difference in intensity. From this observation it may be deduced that neither the application of saline to the injection site nor the topical administration of DMSO to a distant site was effective in preventing tissue damage. However, only 3 animals in group 2 developed lesions at site A. Two of these progressed to 3+ intensity and may have been due to partial administration of Adriamycin i.d. inadvertently, since a double syringe technique (as used in clinical practice for i.v. injections) was not employed. The 3rd animal developed a lesion at site A on the 4th day. It attained + maximal intensity and was healed by Day 7. Accordingly, direct topical application of DMSO to the site of s.c. injection of 2 mg of Adriamycin prevented the appearance of even minimal tissue damage in the majority of animals.

Injection of 0.005 mg of Adriamycin i.d. evoked no lesions within 7 days. A dose of 0.01 mg i.d. produced + lesions in all animals in groups 1 and 2 by one week. Only 50% (4/8) of the animals in Group 3 had such lesions persisting at one week. All animals receiving 0.05 mg of the drug i.d. had 2+ lesions, there being no apparent benefit from the topical application of DMSO within the observation period of 7 days. Thus the ability of DMSO to modify the tissue damage consequent upon i.d. injection of Adriamycin is critically related to the dose of the latter agent.

*Study 2* Each guinea pig was injected with 4 doses of Adriamycin i.d.

A. 0.1 mg at left shoulder.
B. 0.05 mg at left hip.
C. 0.01 mg at right hip.
D. 0.005 mg at right shoulder.

Intervention was accomplished by 3 protocols.

1. Eight animals received DMSO alone at each injection site.
2. Eight animals received DMSO with 10% alpha tocopherol succinate at each injection site.
3. Eight animals received DMSO with 50% alpha tocopherol succinate at each injection site.

The mean scores for the lesions at intervals of 3 days are given in Table II and the statistical analysis is summarized in Table III.

**Table II** Mean scores of tissue damage at intervals of 3 days after i.d. injection of Adriamycin and subsequent topical application of DMSO alone or DMSO with 10 or 50% alpha tocopherol succinate

| | | *Days after injection* | | | | | | |
|---|---|---|---|---|---|---|---|---|
| *Dose* | *Protocol* | *3* | *6* | *9* | *12* | *15* | *18* | *21* |
| | 1 | 2.125 | 3.000 | 4 | 4 | 4 | 4 | 4 |
| A | 2 | 1.750 | 2.875 | 4 | 4 | 4 | 4 | 4 |
| | 3 | 1.250 | 2.625 | 4 | 4 | 4 | 4 | 4 |
| | 1 | 1.875 | 2.375 | 4 | 4 | 4 | 4 | 4 |
| B | 2 | 1.375 | 2.125 | 4 | 4 | 4 | 3.625 | 3.375 |
| | 3 | 0.875 | 1.375 | 4 | 4 | 4 | 3.000 | 3.125 |
| | 1 | 1.000 | 1.250 | 2.125 | 2.875 | 1.625 | 0.625 | 0 |
| C | 2 | 0.125 | 0.625 | 1.500 | 2.125 | 1.375 | 0 | 0 |
| | 3 | 0 | 0.125 | 1.375 | 2.000 | 1.375 | 0 | 0 |
| | 1 | 0 | 0 | 0.750 | 0.875 | 0.500 | 0 | 0 |
| D | 2 | 0 | 0 | 0.500 | 0.250 | 0.250 | 0 | 0 |
| | 3 | 0 | 0 | 0.250 | 0 | 0 | 0 | 0 |

**Table III** Levels of significance (*P* values) in differences between effects of intervention with DMSO or DMSO with 10 or 50% alpha tocopherol succinate for secondary prophylaxis of soft-tissue injury induced by i.d. injection of Adriamycin. NS = Not significant

| | | *Days after injection* | | | | | | |
|---|---|---|---|---|---|---|---|---|
| *Dose* | *Protocol comparison* | *3* | *6* | *9* | *12* | *15* | *18* | *21* |
| | 1 *vs* 2 | NS | NS | NS | NS | NS | NS | NS |
| A | 1 *vs* 3 | NS | NS | NS | NS | NS | NS | NS |
| | 2 *vs* 3 | NS | NS | NS | NS | NS | NS | NS |
| | 1 *vs* 2 | <0.05 | NS | NS | NS | NS | NS | <0.05 |
| B | 1 *vs* 3 | <0.005 | <0.005 | NS | NS | NS | <0.01 | <0.05 |
| | 2 *vs* 3 | NS | <0.005 | NS | NS | NS | NS | NS |
| | 1 *vs* 2 | <0.001 | <0.05 | <0.05 | NS | NS | NS | NS |
| C | 1 *vs* 3 | <0.001 | <0.001 | <0.025 | <0.025 | NS | NS | NS |
| | 2 *vs* 3 | NS | <0.05 | NS | NS | NS | NS | NS |
| | 1 *vs* 2 | NS | NS | NS | <0.025 | NS | NS | NS |
| D | 1 *vs* 3 | NS | NS | <0.025 | <0.005 | <0.05 | NS | NS |
| | 2 *vs* 3 | NS | NS | NS | NS | NS | NS | NS |

Although lesions induced by all doses of Adriamycin evolved more slowly with the topical application of increasing concentrations of alpha tocopherol succinate in DMSO (zero, 10 and 50% in protocols 1, 2 and 3 respectively), lesions with doses A and B were fully developed (4+) in all animals by Day 9. The maximum intensity of soft tissue injury caused by dose C was delayed to Day 12 and it was inversely related to the concentration of alpha tocopherol succinate. A similar, albeit less pronounced effect was seen after dose D, although no lesion was observed in any animal receiving this dose within one week of injection. Lesions following doses C and D resolved completely in all animals and the rate was related directly to the concentration of alpha tocopherol succinate used for intervention. Within 3 weeks the lesions produced by dose B showed early healing only in those animals in which intervention by protocols 2 and 3 was undertaken. By 6 weeks all lesions revealed evidence of healing, although many which had been 4+ in intensity exhibited prominent scar formation.

Following inadvertent extravasation in clinical practice, Adriamycin can be detected in the skin for several months (Garnick *et al.*, 1981). The resulting manifestations of soft-tissue injury may be due to enzymatic reduction of the drug to free radicals (Powis & Appel, 1980) which can form cytotoxic hydroxyl radicals in the presence of molecular oxygen (Fridovich, 1976). These free and hydroxyl radicals may be susceptible to scavenging by alpha tocopherol and dimethyl sulphoxide respectively (Tappel, 1962; Cederbaum *et al.*, 1977). DMSO also facilitates skin penetration (Kligman, 1965) and may disperse Adriamycin from within the skin.

Considerations such as these led Svingen *et al.* (1981) to investigate the roles of DMSO and alpha tocopherol in the secondary prevention of ulceration following i.d. injection of Adriamycin in rats. Their studies demonstrated that the topical application of 10% alpha tocopherol succinate in DMSO at the injection site reduced the mean ulcer diameter.

In our experiments using a guinea pig model, the doses of Adriamycin used were critical. By the i.d. route, 0.01 mg was the minumum amount of the drug which evoked consistent early lesions; a figure identical to that obtained by Rudolph *et al.* (1979). For animals weighing ~250 g, a s.c. dose of 2 mg was a satisfactory compromise between early fatal toxicity and lack of local injury. When the drug was given by the i.d. route we confirmed the value of DMSO plus alpha tocopherol succinate in *reducing* soft tissue injury. Furthermore, this beneficial effect was more evident with the higher concentration of Vitamin E (50%), although it was progressively less with increasing doses of Adriamycin.

As a parallel to clinical circumstances, s.c. injection is a more accurate reflection of inadvertent extravasation since Adriamycin is administered customarily into an established i.v. infusion. Our data on the efficacy of DMSO in preventing soft-tissue injury in most animals following s.c. injection of Adriamycin are thus clinically valid. There was no merit in applying DMSO at a site distant from that at which Adriamycin was injected, as has been suggested by others (Desai *et al.*, 1981). In any event this manoeuvre has no relevance to clinical practice.

DMSO is virtually non-toxic in humans (Rubin, 1975), as is alpha tocopherol in less than mega-doses (Ayres *et al.*, 1979). Although pre-treatment with systemic Vitamin E may potentiate Adriamycin-induced myelosuppression in mice (Alberts *et al.*, 1978), it is unlikely that this will be a factor in the topical use of small amounts during the short-term for the prevention of drug-induced skin necrosis. Therefore we recommend that a solution of 50% alpha tocopherol succinate in DMSO be readily available at all times in locations in which patients are receiving i.v. cytotoxic chemotherapy, especially with Adriamycin, so that it may be applied promptly to any site of suspected extravasation with a view to preventing or at least diminishing the consequent soft tissue injury.

This work was supported by a grant from the Leukemia Research Fund.

### References

ALBERTS, D.S., PENG, Y.-M. & MOON, T.E. (1978). αtocopherol pre-treatment increases Adriamycin bone marrow toxicity. *Biomedicine* (Paris), **29**, 189.

AYRES, S., MIHAN, R. & SCRIBNER, M.D. (1979). Synergism of vitamins A and E with dermatologic applications. *Cutis*, **23**, 600.

BARLOCK, A.L., HOWSER, D.M. & HUBBARD, S.M. (1979). Nursing management of Adriamycin extravasation. *Am. J. Nurs.*, **79**, 94.

BARR, R.D., BENTON, S.G. & BELBECK, L.W. (1981). Soft tissue necrosis induced by extravasated cancer chemotherapeutic agents. *J. Natl Cancer Inst.*, **66**, 1129.

BARR, R.D. & SERTIC, J. (1981). Soft tissue necrosis induced by extravasated cancer chemotherapeutic agents: a study of active intervention. *Br. J. Cancer*, **44**, 267.

CEDERBAUM, A.I., DICKER, E., RUBIN, E. & COHEN, G. (1977). The effect of dimethyl sulphoxide and other hydroxyl radical scavengers on the oxidation of ethanol by rat liver microsomes. *Biochem. Biophys. Res. Commun.*, **78**, 1254.

DESAI, M.H., LORING, L. & TERES, D. (1981). Prevention of Adriamycin-induced skin ulcers in the rat with dimethyl sulphoxide (DMSO) *Proc. Am. Assoc. Cancer Res. and ASCO*, **22**, 70.

FRIDOVICH, I. (1976). Oxygen radicals, hydrogen peroxide and oxygen toxicity. In *Free Radicals in Biology*, Vol. 1, p. 239. (Ed. Pryor) Academic Press, New York.

GARNICK, M., ISRAEL, M., KHETARPAL, V. & LUCE, J. (1981). Persistence of anthracycline levels following dermal and subcutaneous adriamycin extravasation. *Proc. Am. Assoc. Cancer Res. and ASCO*, **22**, 173.

KLIGMAN, A.M. (1965). Topical pharmacology and toxicology of dimethyl sulphoxide—Part 1. *JAMA*, **193**, 796.

LIPPMAN, M., ZAGER, R. & HENDERSON, E.S. (1972). High dose daunorubicin (NSC 83142) in the treatment of advanced acute myelogenous leukemia. *Cancer Chemother. Rep.*, **56**, 755.

POWIS, G. & APPEL, P.L. (1980). Relationship of the single electron reduction potential of quinones to their reduction by flavoproteins. *Biochem. Pharmacol.*, **29**, 2567.

RUBIN, L.F. (1975). Toxicity of dimethyl sulphoxide, alone and in combination. *Ann. N.Y. Acad. Sci.*, **243**, 98.

RUDOLPH, R., SUZUKI, M. & LUCE, J.K. (1979). Experimental necrosis produced by Adriamycin. *Cancer Treat. Rep.*, **63**, 529.

SVINGEN, B.A., POWIS, G., APPEL, P.L. & SCOTT, M. (1981). Protection against Adriamycin-induced skin necrosis in the rat by dimethyl sulphoxide and αtocopherol. *Cancer Res.*, **41**, 3395.

TAPPEL, A.L. (1962). Vitamin E as the biological lipid antioxidant. *Vitam. Horm.*, **20**, 493.